# Mismatches in 16S rRNA Gene Primers: An Area Worth Further Exploring

**DOI:** 10.3389/fmicb.2022.888803

**Published:** 2022-06-13

**Authors:** Wenting Ren, Yingwen Zhong, Yi Ding, Yuehong Wu, XueWei Xu, Peng Zhou

**Affiliations:** ^1^Key Laboratory of Marine Ecosystem Dynamics, Ministry of Natural Resources and Second Institute of Oceanography, Ministry of Natural Resources, Hangzhou, China; ^2^School of Oceanography, Shanghai Jiao Tong University, Shanghai, China

**Keywords:** metagenomic sequencing, 16S rRNA gene amplicon sequencing, universal primers, mismatch, microbiome

## Introduction

The previous study has shown that ~10% of environmental microbial sequences might be missed from classical PCR-based SSU rRNA gene surveys, and primer mismatches would probably significantly reduce or prevent the recovery of taxonomic “blind spots” in PCR-based surveys (Eloe-Fadrosh et al., 2016). In spite of its deficiency, currently, 16S rRNA gene amplicon sequencing remains widely used in the studies on microbial communities (Liu et al., 2019; Zhang et al., 2019; Deng et al., 2020; Kitamoto et al., 2020; Gonzalez et al., 2021). The literature on 16S rRNA gene amplicon showed a significant upward trend based on the search against PubMed (https://pubmed.ncbi.nlm.nih.gov) (Supplementary Figure 1). However, in 2021, Palkova et al. reported that certain different primer sets toward 16S rRNA gene amplicon sequencing could provide rather opposite Bacteroidetes/Firmicutes ratio while investigating the outcome of sequencing analysis on intestinal microbiota from children with autism spectrum disorder (Palkova et al., 2021). Currently, the microbiome, as a potential diagnostic and predictive biomarker in severe alcoholic hepatitis, has been surveyed by 16S rRNA gene amplicon sequencing (Kim et al., 2021), to which coverage and accuracy are very important. It is necessary to draw attention to defects in 16S rRNA gene amplicon sequencing and call for further exploring mismatches in 16S rRNA gene primers, especially for diagnosis by surveying the microbiome.

## Primer Mismatches

The 16S rRNA gene sequences mismatched with 18 frequently used bacterial universal primers (details shown in Supplementary Table 1) were screened by using BLASTN (e-value ≤ 0.001) against 592,605 bacteria rRNA gene sequences in the SILVA SSURef_NR99 database (release 132, https://www.arb-silva.de), which provides comprehensive, quality checked, and regularly updated datasets of aligned rRNA sequences. For each primer, the number (percentage) of mismatched sequences and the top three families with mismatched sequences are shown in Table 1. Among the surveyed forward and reverse primers, 515F and U529R had the lowest percentage of mismatch, 1.08 and 0.79%, respectively (shown in Table 1). There are 14 complimentary nucleotides overlapped between the forward primer 515F and reverse primer U529R (Supplementary Table 1), which explains similar taxa with mismatches to primers 515F and U529R, such as that the family *Lachnospiraceae* showed a high percentage of mismatch to primers 515F (0.06%) and U529R (0.04%), as shown in Table 1. Outside the 14 complimentary nucleotides, there are fewer mismatches to U529R (0.021%) than those to 515F (0.031%), which resulted in a lower percentage of mismatch to U529R in *Lachnospiraceae* (0.04%). Moreover, the family *Lachnospiraceae* had a high percentage of mismatches with primers U341F, 515F, 517F, 338R, U529R, 533R, and 907R (Table 1). The family *Lachnospiraceae* belongs to the core of gut microbiota, and its abundance was associated with aging (Odamaki et al., 2016), within which specific taxa were involved in different intra- and extra-intestinal diseases (Vacca et al., 2020). Other families closely related to human health and disease, *Propionibacteriaceae, Bacillacea, Burkholderiaceae, Staphylococcaceae*, and *Veillonellaceae*, showed highly mismatched rates as well. For example, certain species of the family *Propionibacteriaceae* were considered potential pathogens in acne and other skin conditions (Berman, 2012).

**Table 1:** The number (percentage) of mismatched sequences to 18 universal primers against the SILVA bacteria database.

**Direction**	**Primer**	**Number of mismatched sequences (%)**	**Top three families with mismatched sequences (%)**
Forward	515F	6 190 (1.08%)	*Lachnospiraceae* (0.06%) *Bacillaceae* (0.06%) *Burkholderiaceae* (0.05%)
	517F	23 326 (4.00%)	*Propionibacteriaceae* (0.52%) *Lachnospiraceae* (0.16%) *Staphylococcaceae* (0.13%)
	U341F	33 579 (5.83%)	*Anaerolineaceae* (0.36%) *Lachnospiraceae* (0.31%) *Chthoniobacteraceae* (0.13%)
	1099F	55 641 (9.82%)	*Flavobacteriaceae* (1.49%) *Prevotellaceae* (1.19%) *Bacteroidaceae* (0.97%)
	784F	92 495 (15.93%)	*Propionibacteriaceae* (0.59%) SAR11-Clade I (0.50%) *Veillonellaceae* (0.46%)
	909F	134 448 (23.06%)	*Enterobacteriaceae* (6.29%) *Pseudomonadaceae* (3.36%) *Moraxellaceae* (2.09%)
	8F	62 814 (25.99%)	NA
	27F	65 077 (27.16%)	NA
	967F	211 987 (36.90%)	*Burkholderiaceae* (5.38%) *Rhodobacteraceae* (2.11%) *Rhizobiaceae* (1.59%)
Reverse	U529R	4 490 (0.79%)	*Staphylococcaceae* (0.04%) *Bacillaceae* (0.04%) *Lachnospiraceae* (0.04%)
	533R	27 471 (4.69%)	*Propionibacteriaceae* (0.57%) *Lachnospiraceae* (0.21%) *Staphylococcaceae* (0.16%)
	338R	38 017 (6.58%)	*Anaerolineaceae* (0.36%) *Lachnospiraceae* (0.35%) *Enterobacteriaceae* (0.17%)
	806R	42 881 (7.35%)	*Propionibacteriaceae* (0.60%) SAR11-Clade I (0.49%) *Microbacteriaceae* (0.40%)
	907R	48 108 (8.24%)	*Lachnospiraceae* (0.34%) *Bacillaceae* (0.30%) *Sphingomonadaceae* (0.30%)
	1046R	50 357 (8.61%)	*Burkholderiaceae* (0.75%) *Rhodothermaceae* (0.38%) *Veillonellaceae* (0.30%)
	1391R	43 530 (9.35%)	NA
	798R	64 482 (11.40%)	*Veillonellaceae* (0.47%) *Pirellulaceae* (0.44%) *Cyanobiaceae* (0.31%)
	1492R	114 489 (51.99%)	NA

*The sequences and references of the universal primers are provided in Supplementary Table 1. Percentage of mismatched sequences to universal primers (%) = {number of mismatched sequences / number of sequences hit by primer} × 100%. NA, no data available*.

*Different families may have a different diversity representation. Taking primer 515F, for example, the top three families found with the most mismatches were Lachnospiraceae (0.06%), Bacillaceae (0.06%), and Burkholderiaceae (0.05%). Normalized by the sequence number of Lachnospiraceae, Bacillaceae, and Burkholderiaceae in the SILVA database, the percentage of mismatched sequences within the family was 0.93 (362/38,850), 1.76 (342/19,464), and 0.91% (289/31,828), respectively*.

## Case Study on Primer 515F

Therefore, to emphasize that even the primer 515F, which showed the lowest percentage of mismatch (1.08%) against the SILVA bacteria database, may have significant effects on certain taxa due to primer mismatch in analyzing microbial community composition using 16S rRNA gene amplicons, six sequencing datasets on three stool samples from the human gastrointestinal tract, including both 16S rRNA gene amplicon sequencing and metagenomic sequencing (Peters et al., 2019), were chosen for further investigation. Three stool samples (S1, S2, and S3) were collected from patients with melanoma receiving different times for immunotherapy. The three immunotherapy times of S1, S2, and S3 were baseline, week 6, and week 12, respectively. The metagenomic datasets for S1, S2, and S3 were named 1-M, 2-M, and 3-M, respectively, whereas the amplicon datasets were named 1-A, 2-A, and 3-A. Details about datasets are available in Supplementary Table 2. Estimated by Nonpareil software, the coverage of the actual sequencing depth for the three metagenomic datasets 1-M, 2-M, and 3-M was 0.99, 0.98, and 0.95, respectively, indicating that sufficient sequencing depth was achieved for further analysis (*C* = 0.95, as a rule-of-thumb for nearly complete coverage) (Rodriguez and Konstantinidis, 2014; Rodriguez-R et al., 2018). The estimated coverage curves for these datasets are shown in Supplementary Figure 2.

Afterward, we compared the composition of the microbial community *via* the two sequencing methods and the histograms of relative abundance at the family level for six datasets shown in Supplementary Figure 3. The results showed that *Bacteroidaceae* was dominant in all six datasets. The relative abundance of this family in the datasets 1-M, 2-M, and 3-M was significantly higher than those in the datasets 1-A, 2-A, and 3-A (Supplementary Figure 4 and Supplementary Table 2). Also, the relative abundance of *Lachnospiraceae, Ruminococcaceae, Enterobacteriaceae*, and *Fusobacteriaceae* was obviously higher in datasets 1-M, 2-M, and 3-M, when compared to datasets 1-A, 2-A, and 3-A, respectively (Supplementary Figure 4 and Supplementary Table 2). Consistently, certain species of *Bacteroidaceae* and *Lachnospiraceae* were not detected, either at all or with sufficient abundance in the 16S rRNA gene amplicon sequencing datasets denoted with Greengenes Database (Peters et al., 2019).

Since the universal primer 515F was used for 16S rRNA gene amplicons, bacterial 16S rRNA gene reads covering the primer 515F region were screened in the metagenomic datasets, 37,028 in 1-M, 41,178 in 2-M, and 36,999 in 3-M, respectively (refer to Supplementary Methods for details). Notably, the numbers of reads mismatched to primer 515F in the datasets 1-M, 2-M, and 3-M were 4,619, 6,627, and 6,343, respectively, and the percentage of mismatched reads to primer 515F (PMR-515F) was 12.47 (4,619/37,028), 16.09 (6,627/41,178), and 17.14% (6,343/36,999) (Supplementary Table 4). Furthermore, PMR-515F for taxa (relative abundance > 0.04%) in the datasets1-M, 2-M, and 3-M was analyzed. The family *Bacteroidaceae* showed the highest PMR-515F in the datasets 1-M, 2-M, and 3-M (7.51, 8.34, and 5.40%, respectively) (Supplementary Figure 4), which could be one of the possible explanations for its higher relative abundance in the metagenomic datasets than those in the corresponding amplicon datasets. If reads with mismatch to 515F were excluded from the metagenomic datasets, the relative abundance was closer to that in amplicon datasets (Supplementary Figure 4). Similarly, PMR-515F of the family *Lachnospiraceae* in the datasets 1-M, 2-M, and 3-M were 0.90, 2.78, and 3.48%, respectively (Supplementary Figure 4), which may result in the higher relative abundance in the metagenomic datasets compared with the amplicon datasets. It was consistent with the above result of *Lachnospiraceae* as one of the taxa with the most mismatched sequences by aligning primer 515F against the SILVA bacteria database. Besides *Lachnospiraceae*, some other families also showed relatively high PMR-515F in the metagenomic datasets, such as 3.34% for *Ruminococcaceae* in 3-M, 1.87% for *Fusobacteriaceae* in 1-M, 1.83% for *Enterobacteriaceae* 2-M, and 1.95% for *Tannerellaceae* in 3-M (Supplementary Figure 4).

Furthermore, the reads mismatched to 515F in the top three families in the metagenomic datasets were analyzed at genus and species levels, and the genus *Bacteroides* showed high PMR-515F (details in Supplementary Table 5). For instance, in all three metagenomic datasets, some reads with mismatched sequences to primer 515F were found as segments of the 16S rRNA gene of *Bacteroides vulgatus*, (2 reads in 1-M, 13 reads in 2-M, and 7 reads in 3-M), whereas the downstream sequence of the primer 515F in those reads was not detected in the corresponding amplicon datasets using BLASTN. Similarly, some reads with mismatched sequences to primer 515F were annotated as segments of the 16S rRNA gene of *B. thetaiotaomicron* in the metagenomic datasets (3, 18, and 4), none of which were detected in the amplicon datasets. *B. vulgatus* and *B. thetaiotaomicron* were opportunistic pathogens, which could induce severe colitis. The results suggested that primer mismatches have an effect on the accuracy of detecting pathogenic bacteria in the 16S rRNA gene amplicon sequencing. Furthermore, to evaluate underestimation in amplicon sequencing at a higher level, the intra-family PMR-515F in the metagenomic datasets was investigated (shown in Supplementary Figure 5). The results showed significant intra-family PMR-515F for *Bacteroidaceae* (11.85% in 1-M, 15.74% in 2-M, and 16.89% in 3-M), *Tannerellaceae* (13.24% in 1-M, 47.06% in 2-M, and 17.58% in 3-M), *Lachnospiraceae* (12.16% in 1-M, 15.81% in 2-M, and 15.95% in 3-M), and *Ruminococcaceae* (14.19% in 1-M, 16.10% in 2-M, and 16.26% in 3-M). Consistent with the previous study, metagenomic sequencing could uncover a more comprehensive composition of microorganisms in the environment, including the microbial groups that were underestimated or ignored in the analysis of amplicon sequencing (Eloe-Fadrosh et al., 2016).

## Summary

This study analyzed the primer mismatches from the SILVA database to the experimental datasets. The case study showed the effects of amplicon on the composition of a microbial community. Here, the importance of an approach with less bias is emphasized for the studies on a microbial community. Since the microbiome could be considered the potential diagnostic biomarker (Kim et al., 2021), the accuracy of inferred microbial community composition is essential for diagnosis. With the development in sequencing technology, the methods, which do not require sequence-dependent primer annealing, should be applied more extensively.

## Author Contributions

PZ: study concept and design. WR: acquisition of data and statistical analysis. WR, YZ, and PZ: analysis and interpretation of data. WR, YW, PZ, and XX: drafting and editing of the manuscript. WR and YD: manuscript revision. XX: study supervision. All authors contributed to the article and approved the submitted version.

## Funding

This work was supported by grants from the National Science and Technology Fundamental Resources Investigation Program of China (2021FY100900), the Oceanic Interdisciplinary Program of Shanghai Jiao Tong University (No. SL2020MS027), Scientific Research Fund of the Second Institute of Oceanography, MNR (No. JZ1901), and Fund for International Cooperation, State Oceanic Administration of China (Nos. 17070393 and 18070323).

## Conflict of Interest

The authors declare that the research was conducted in the absence of any commercial or financial relationships that could be construed as a potential conflict of interest.

## Publisher's Note

All claims expressed in this article are solely those of the authors and do not necessarily represent those of their affiliated organizations, or those of the publisher, the editors and the reviewers. Any product that may be evaluated in this article, or claim that may be made by its manufacturer, is not guaranteed or endorsed by the publisher.
