# Supplementary material for: Mismatches in 16S rRNA Gene Primers: An Area Worth Further Exploring
Source: Front Microbiol. 2022 Jun 13;13:888803. doi: 10.3389/fmicb.2022.888803 (PMC9234566; doi:10.3389/fmicb.2022.888803)
Supplement: Supplementary file 1 [file Data_Sheet_1.docx]

# Supplementary materials

## Supplementary Methods

### *1.1 Analysis of 18 universal primes against SILVA database*

The SILVA Bacteria Database was performed, based on the FASTA profile of non-redundant SILVA SSURef_NR99 database (release 132, <https://www.arb-Silva.de/no_cache/download/archive/release_132/Exports>). 18 universal bacteria primers were chosen (**Supplementary Table S1**) and mapped to the SILVA Bacteria Database by using Blastn with an e-value cutoff of 0.001 and word_size of 4, the result profiles were considered to be hit rRNA sequences with each primer. Matched sequences hit by each primer were defined that all sequences for permutation and combination of the degenerate base by using SEQKIT software (Shen et al., 2016). Excluding the matched sequences, the rest of primer sequences by Blastn were considered as mismatched sequences hit by each primer (universal primer 515F as an example in **Supplementary Figure S6**). Based on the profiles of the SILVA accession, the FASTA profile of mismatched sequences and their taxa were extracted by SEQKIT software and in-house shell scripts. The taxonomic hierarchy of mismatched sequences was received by the criterion that meant unclassified information of taxonomic hierarchy was defined uniformly as “unidentified”.

### *1.2 Selection of samples*

Six datasets were selected from the studies using both 16S rRNA gene amplicon sequencing by primer 515F (5’-GTGCCAGCMGCCGCGG-3’) and metagenomic sequencing. These samples were collected from stool with melanoma patients (Peters et al., 2019). Data are available in the NCBI Short Read Archive (SRA) database (<https://www.ncbi.nlm.nih.gov/sra/>). The accession numbers for metagenomic sequencing were SRR9033715 (dataset 1-M), SRR9033758 (dataset 2-M) and SRR9033757 (dataset 3-M), the accession numbers for amplicon sequencing were SRR9207278 (dataset 1-A), SRR9207283 (dataset 2-A) and SRR9207307 (dataset 3-A), respectively (**Supplementary Table S2**).

The SRA format of six datasets was transferred to FASTQ files by fastq-dump tool (<https://github.com/ncbi/sra-tools/tree/master/tools/fastq-dump>) with the NCBI SRA toolkit package (version 2.9.1) (<https://github.com/ncbi/sra-tools>). The FASTQ files were handled by Fastp software (version 0.20.1), to obtain the clean data with high-quality reads (Chen et al., 2018). Quality control was analyzed by FastQC software (version 0.11.9) (Andrews, 2010). The high-quality reads were converted FASTQ to FASTA format by using SEQKIT software (Shen et al., 2016).

The coverage of microbial community for datasets 1-M, 2-M and 3-M were estimated by bioinformatic software Nonpareil (Rodriguez and Konstantinidis, 2014; Rodriguez-R et al., 2018). The coverages for datasets 1-M, 2-M and 3-M were 0.99, 0.98 and 0.95, respectively, which satisfied the threshold of 0.95. Projected sequencing effort for nearly complete coverage were 1,834,802,793 bp, 6,823,248,170 bp and 6,823,248,170 bp, the sequence diversities were 17.8, 17.5 and 18.8, respectively (**Supplementary Figure S2**). Thus, the biases due to the sequencing depth were not considered in this study.

## *1.3 Analysis of 16S sequences from amplicon and metagenome*

Comparing to 16S rRNA gene amplicon sequencing and assembled metagenomes, short sequencing reads in the metagenomes contained more sufficient information to reflect the composition of species and functional annotation in the microbial community (Carr and Borenstein, 2014; Berlemont et al., 2020). The 16S rRNA gene reads were extracted by SortMeRNA software (version 4.3.2) from the datasets 1-M, 2-M and 3-M, directly mapping the SILVA SSURef_NR99 database with threshold value 97% (Kopylova et al., 2012), and then they were filtered through the length more than 80 bp and less than 150 bp by SEQKIT software (Shen et al., 2016). The filtered metagenomic 16S rRNA gene reads for each dataset were combined into one FASTA file and repeated reads id were renamed to make them unique. Reads hit by primer 515F were obtained via using 16S rRNA gene reads in each datasets 1-M, 2-M and 3-M were mapped to the SILVA Bacteria Database by Blastn with an e-value cutoff of 0.001 and word_size of 4. In the datasets 1-A, 2-A and 3-A, only the reads amplified by primer 515F were used. The taxa of 16S rRNA gene reads were annotated by using Blastn against the SILVA Bacteria Database from domain to family levels, to analyze the relative abundance at the family level in the six datasets. The results were visualized by using Origin software.

Matching reads to primer 515F in the 16S rRNA gene reads from each dataset 1-M, 2-M and 3-M was performed by using the function of locating subsequences/motifs in the SEQKIT software (Shen et al., 2016). Mismatched reads to primer 515F were obtained via eliminating the matched reads to primer 515F in the metagenomic 16S rRNA reads. And then FASTA files of mismatched reads to primer 515F were obtained by a python script. To figure out the taxa of mismatched reads, mismatched reads to primer 515F were annotated by using Blastn against the SILVA Bacteria Database. According to taxonomic analysis, the taxa that were significantly enriched in the different datasets were described. In addition, PMR-515F was calculated based on the numbers of mismatched reads to primer 515F, PMR-515F (%) = {number of mismatched reads hit by 515F / number of reads hit by 515F} × 100%. As the primer and barcode of the amplicon raw data was already cut off, the percent and taxa of mismatches could not be compared and analyzed with metagenomic 16S rRNA. And unless otherwise stated, the comparison of results was routinely performed through the reads hit by the SILVA Bacteria Database or primer 515F in the datasets 1-M, 2-M and 3-M.

**Supplementary Figure S1** Literature on 16S rRNA gene amplicon in PubMed for the last ten years. Solid and dashed lines represented search results for “16S rRNA gene amplicon” and “gut 16S rRNA gene amplicon”, respectively.


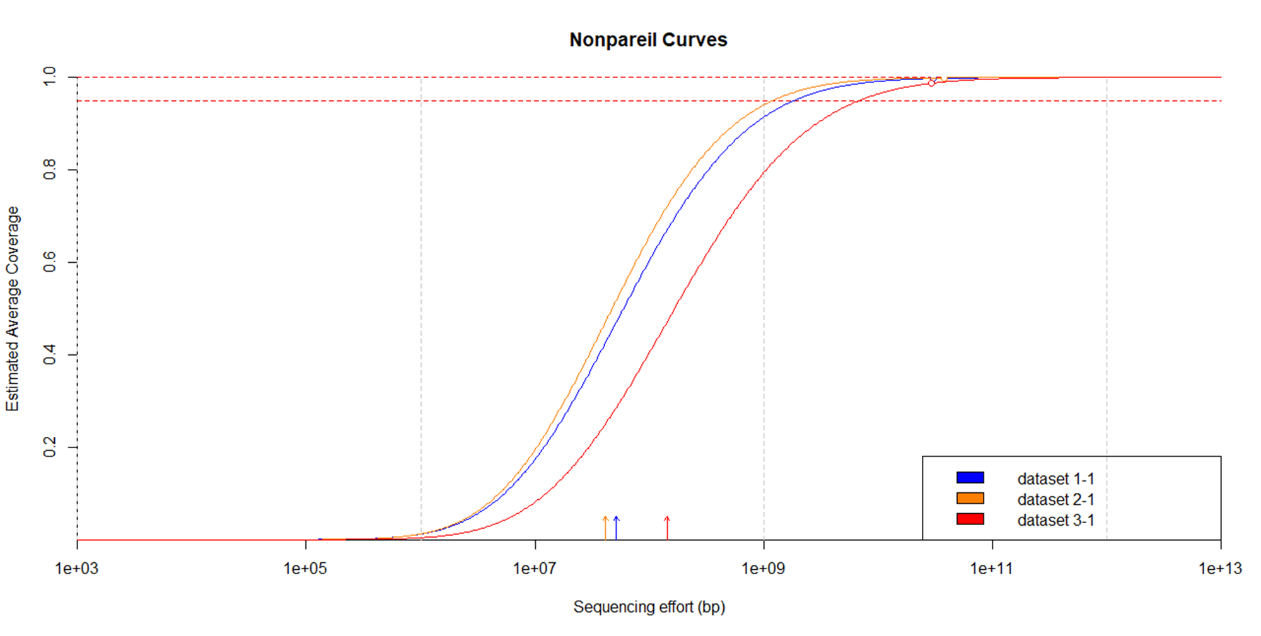


**Supplementary Figure S2** Curves for the estimated coverage of the gut microbial communities with three metagenomics datasets. The red horizontal dashed lines represent 1 and 0.95 coverage. Circles on curves represent the coverage of the actual sequencing depth for each dataset in relation to the entire curve.

**Supplementary Figure S3** Relative abundance of bacterial communities at family level based on metagenomic short reads (80-150 bp) hit by primer 515F and 16S rRNA amplicon reads amplified with primer 515F (>=150 bp). It mainly showed that individual family abundance was higher than 0.4% in the dataset. The legend ‘others’ represented that the family abundance was below 0.4%.

**Supplementary Figure S4** Relative abundance (primary *y*-axis) and percentage of mismatched reads (secondary *y*-axis) of nine families in the six datasets. S1, S2 and S3 on *x-axis* represented for sample1, sample2 and sample3, respectively. Solid, dashed and dotted lines represented relative abundance at family level. Grey column represented percentage of mismatched reads to primer 515F in the metagenomic datasets.

**Supplementary Figure S5** The PMR-515F within the family in the three metagenomic datasets. PMR-515F within the family was calculated based on the formula, {number of mismatched reads within the family hit by 515F / number of reads within the family hit by 515F) × 100%. It mainly showed that individual family abundance was higher than 0.4% in the datasets.


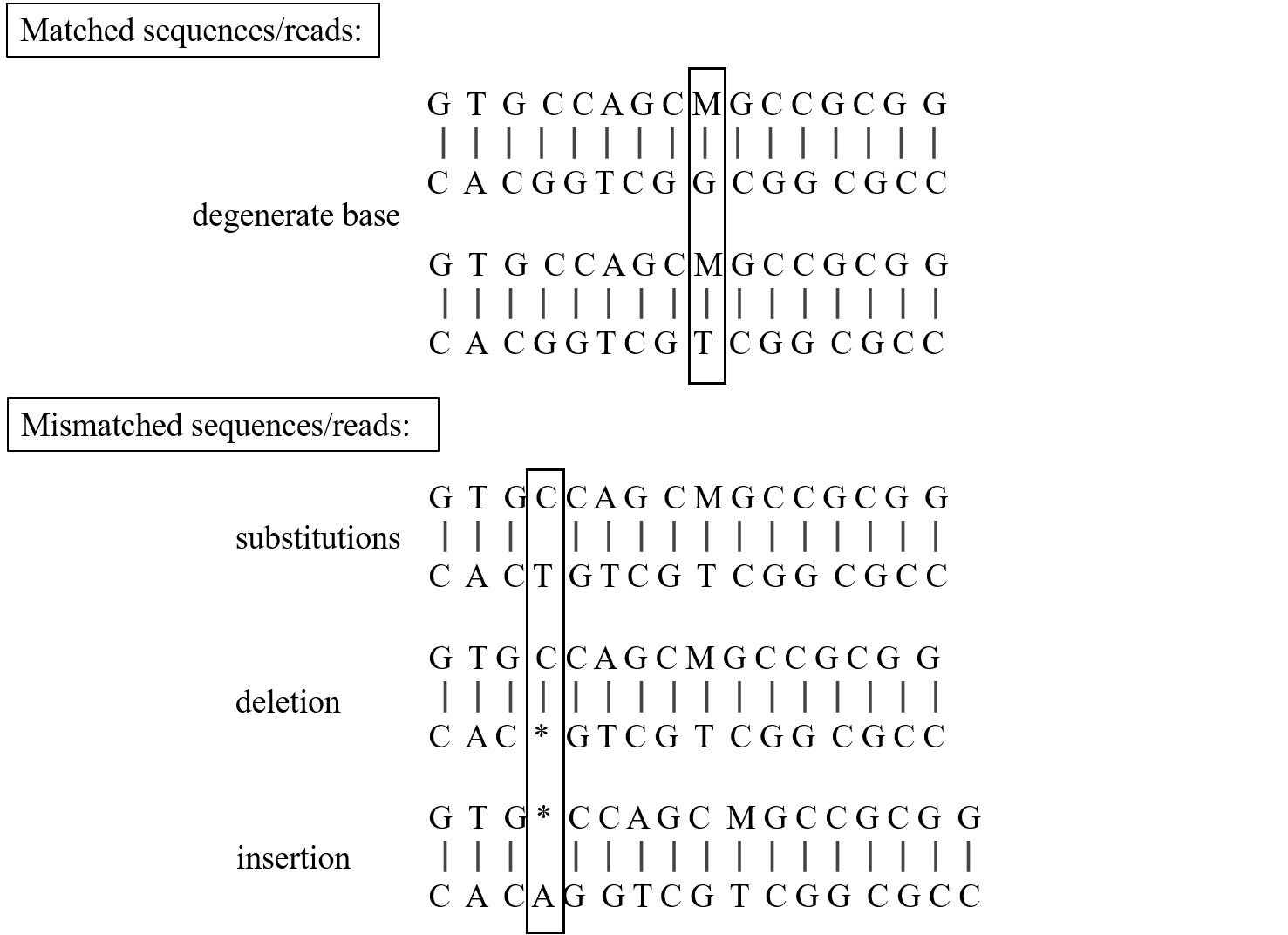


**Supplementary Figure S6** The example of matched / mismatched sequences / reads for universal primer 515F.

**Supplementary Table S1** The detailed information for universal primers used in this study.

| Direction | Primer | Sequence 5'-3'^*^ | Cites |
| --- | --- | --- | --- |
| forward | 8F | AGAGTTTGATYMTGGCTCAG | (Juretschko et al., 1998; Kim et al., 2009) |
|  | 27F | AGAGTTTGATCMTGGCTCAG | (Suzuki and Giovannoni, 1996; Hongoh et al., 2003) |
|  | U341F | CCTACGGGRSGCAGCAG | (Baker et al., 2003) |
|  | 515F | GTGCCAGCMGCCGCGG | (Chen et al., 2020) |
|  | 517F | GCCAGCAGCCGCGGTAA | (Wang and Qian, 2009) |
|  | 784F | AGGATTAGATACCCTGGTA | (Andersson et al., 2008) |
|  | 909F | AAACTYAAARRAATTGACGG | (Keijser et al., 2008) |
|  | 967F | CAACGCGAAGAACCTTACC | (Sogin et al., 2006) |
|  | 1099F | GYAACGAGCGCAACCC | (Nossa et al., 2010) |
| reverse | 338R | TGCTGCCTCCCGTAGGAGT | (Fierer et al., 2008) |
|  | U529R | ACCGCGGCKGCTGGC | (Baker et al., 2003) |
|  | 533R | TTACCGCGGCTGCTGGCAC | (Huse et al., 2008) |
|  | 798R | AGGGTATCTAATCCT | (Nossa et al., 2010) |
|  | 806R | GGACTACHVGGGTATCTAAT | (Walters et al., 2011) |
|  | 907R | CCGTCAATTCMTTTGAGTTT | (Muyzer et al., 1995) |
|  | 1046R | CGACARCCATGCASCACCT | (Dethlefsen et al., 2008) |
|  | 1391R | GACGGGCGGTGTGTRCA | (Lane et al., 1985) |
|  | 1492R | TACGGYTACCTTGTTACGACTT | (Lane, 1991) |

^*^Sequence in IUPAC code: R, A/G; Y, C/T; K, G/T; S, G/C; M, A/C; H, A/T/C; V, G/A/C.

**Supplementary Table S2** The information of six gut datasets. Reads numbers represented the numbers of metagenomic 16S rRNA reads extracted by SortMeRNA from paired end clean data and the numbers of amplicon reads amplified by 515F, respectively.

| Sample | Time^*^ | Dataset | SRA accession | Sequencing methods | reads numbers |
| --- | --- | --- | --- | --- | --- |
| Sample1 | Baseline | Dataset 1-M | SRR9033715 | metagenome | 1,141,107 |
|  |  | Dataset 1-A | SRR9207278 | amplicon | 69,292 |
| Sample2 | Week 6 | Dataset 2-M | SRR9033758 | metagenome | 1,274,521 |
|  |  | Dataset 2-A | SRR9207283 | amplicon | 58,649 |
| Sample3 | Week 12 | Dataset 3-M | SRR9033757 | metagenome | 1,088,032 |
|  |  | Dataset 3-A | SRR9207307 | amplicon | 70,018 |

^*^Time was from start of immunotherapy to first progression or death.

**Supplementary Table S3** Relative abundance (>=0.4%) of bacterial communities at family level based on metagenomic reads hit by primer 515F and 16S rRNA amplicon reads amplified with primer 515F for the six datasets.

| Family | Dataset 1-M | Dataset 1-A | Dataset 2-M | Dataset 2-A | Dataset 3-M | Dataset 3-A |
| --- | --- | --- | --- | --- | --- | --- |
| *Bacteroidaceae* | 23,472 (63.39%) | 30,982 (44.71%) | 21,825 (53%) | 20,462 (34.89%) | 11,838 (32%) | 16,181 (23.11%) |
| *Fusobacteriaceae* | 4,225 (11.41%) | 1,750 (2.53%) | 0 (0%) | 0 (0%) | 1 (0%) | 2 (0%) |
| *Lachnospiraceae* | 2,754 (7.44%) | 3,876 (5.59%) | 7,247 (17.6%) | 7,435 (12.68%) | 8,083 (21.85%) | 11,383 (16.26%) |
| *Tannerellaceae* | 1,344 (3.63%) | 2,073 (2.99%) | 17 (0.04%) | 11 (0.02%) | 4,102 (11.09%) | 4,994 (7.13%) |
| *Enterobacteriaceae* | 972 (2.63%) | 1,871 (2.7%) | 4,605 (11.18%) | 3,336 (5.69%) | 282 (0.76%) | 494 (0.71%) |
| *Ruminococcaceae* | 768 (2.07%) | 1,574 (2.27%) | 4,030 (9.79%) | 5,668 (9.66%) | 7,600 (20.54%) | 12,375 (17.67%) |
| *Muribaculaceae* | 562 (1.52%) | 630 (0.91%) | 1 (0%) | 5 (0.01%) | 4 (0.01%) | 5 (0.01%) |
| *Burkholderiaceae* | 485 (1.31%) | 1,172 (1.69%) | 15 (0.04%) | 21 (0.04%) | 326 (0.88%) | 769 (1.1%) |
| *Rikenellaceae* | 475 (1.28%) | 1,083 (1.56%) | 797 (1.94%) | 1,411 (2.41%) | 1,288 (3.48%) | 2,563 (3.66%) |
| *Acidaminococcaceae* | 310 (0.84%) | 406 (0.59%) | 0 (0%) | 1 (0%) | 319 (0.86%) | 285 (0.41%) |
| *Rhodospirillales-uncultured* | 281 (0.76%) | 1,119 (1.61%) | 0 (0%) | 0 (0%) | 0 (0%) | 1 (0%) |
| *Prevotellaceae* | 268 (0.72%) | 410 (0.59%) | 6 (0.01%) | 4 (0.01%) | 45 (0.12%) | 35 (0.05%) |
| *Erysipelotrichaceae* | 188 (0.51%) | 236 (0.34%) | 107 (0.26%) | 157 (0.27%) | 152 (0.41%) | 342 (0.49%) |
| *Christensenellaceae* | 117 (0.32%) | 278 (0.4%) | 26 (0.06%) | 49 (0.08%) | 789 (2.13%) | 1,022 (1.46%) |
| *Akkermansiaceae* | 110 (0.3%) | 423 (0.61%) | 0 (0%) | 3 (0.01%) | 14 (0.04%) | 59 (0.08%) |
| *Desulfovibrionaceae* | 96 (0.26%) | 303 (0.44%) | 144 (0.35%) | 513 (0.87%) | 245 (0.66%) | 613 (0.88%) |
| *Barnesiellaceae* | 79 (0.21%) | 103 (0.15%) | 15 (0.04%) | 3 (0.01%) | 4 (0.01%) | 1 (0%) |
| *Streptococcaceae* | 20 (0.05%) | 2 (0%) | 7 (0.02%) | 2 (0%) | 705 (1.91%) | 526 (0.75%) |
| *Clostridiales-Family XIII* | 20 (0.05%) | 27 (0.04%) | 27 (0.07%) | 28 (0.05%) | 67 (0.18%) | 101 (0.14%) |
| *Bifidobacteriaceae* | 4 (0.01%) | 1 (0%) | 156 (0.38%) | 213 (0.36%) | 40 (0.11%) | 49 (0.07%) |
| *Peptostreptococcaceae* | 4 (0.01%) | 2 (0%) | 56 (0.14%) | 155 (0.26%) | 15 (0.04%) | 29 (0.04%) |
| *Lactobacillaceae* | 0 (0%) | 1 (0%) | 1,417 (3.44%) | 1,145 (1.95%) | 4 (0.01%) | 4 (0.01%) |
| *Veillonellaceae* | 0 (0%) | 0 (0%) | 432 (1.05%) | 629 (1.07%) | 397 (1.07%) | 545 (0.78%) |
| *Aeromonadaceae* | 0 (0%) | 0 (0%) | 23 (0.06%) | 0 (0%) | 0 (0%) | 0 (0%) |

**Supplementary Table S4** The situation of hit matched / mismatched reads with primer 515F for the three gut metagenomic datasets.

|  | Dataset 1-M | Dataset 2-M | Dataset 3-M |
| --- | --- | --- | --- |
| Number of reads hit by 515F | 37,028 | 41,178 | 36,999 |
| Number of matched reads hit by 515F | 32,409 | 34,551 | 30,656 |
| Number of mismatched reads hit by 515F | 4,619 | 6,627 | 6,343 |
| PMR-515F (%) | 12.47% | 16.09% | 17.14% |

PMR-515F (%) = number of mismatched reads hit by 515F / number of reads hit by 515F *100%

**Supplementary Table S5** The taxonomic information of mismatched reads in the datasets 1-M, 2-M and 3-M.

Note: only display top three families / genus with mismatched reads, and the specific species in the Table. Others, non-specific species, such as “uncultured organism”.

|  | Family (PMR-515F %) | Genus (PMR-515F %) | Species |
| --- | --- | --- | --- |
| Dataset 1-M | *Bacteroidaceae* (7.51%) | *Bacteroides* (7.51%) | *Bacteroides dorei*; *Bacteroides salyersiae*; *Bacteroides massiliensis* dnLKV3; *Bacteroides thetaiotaomicron*; *Bacteroides salyersiae*; *Bacteroides caccae*; *Bacteroides uniformis*; *Bacteroides vulgatus*; others |
|  | *Fusobacteriaceae* (1.87%) | *Cetobacterium* (1.85%) | others |
|  |  | *Fusobacterium* (0.02%) | others |
|  | *Lachnospiraceae* (0.90%) | *Ruminococcus gnavus* group (0.18%) | others |
|  |  | *Lachnospiraceae* NK4A136 group (0.14%) | others |
|  |  | *Blautia* (0.14%) | others |
| Dataset 2-M | *Bacteroidaceae* (8.34%) | *Bacteroides* (8.34%) | *Bacteroides dorei*; *Bacteroides thetaiotaomicron*; *Bacteroides vulgatus*; *Bacteroides salyersiae*; *Bacteroides fragilis; Bacteroides caccae; Bacteroides acidifaciens; Bacteroides uniformis; Bacteroides clarus; Bacteroides massiliensis; Bacteroides neonati*; *Bacteroides* sp. R2F3-3-3; others |
|  | *Lachnospiraceae* (2.78%) | *Roseburia* (0.94%) | *Roseburia* sp. 831b; others |
|  |  | *Agathobacter* (0.88%) | others |
|  |  | *Blautia* (0.19%) | others |
|  | *Enterobacteriaceae* (1.83%) | *Enterobacter* (0.75%) | *Enterobacter cloacae*; *Enterobacter* *asburiae*; *Enterobacter mori*; *Enterobacter ludwigii*; *Enterobacter* sp.; others |
|  |  | *Serratia* (0.30%) | *Serratia proteamaculans*; *Serratia symbiotica*; *Serratia plymuthica*; others |
|  |  | *Buttiauxella* (0.23%) | others |
| Dataset 3-M | *Bacteroidaceae* (5.40%) | *Bacteroides* (5.4%) | *Bacteroides salyersiae*; *Bacteroides vulgatus*; *Bacteroides finegoldii; Bacteroides dorei*; *Bacteroides caccae*; *Bacteroides massiliensis*; *Bacteroides stercoris*; *Bacteroides thetaiotaomicron*; *Bacteroides uniformis*; *Bacteroides helcogenes*; *Bacteroides stercoris*; *Bacteroides fragilis*; *Bacteroides* sp. Marseille-P3108; others |
|  | *Lachnospiraceae* (3.48%) | *Eubacterium* *eligens* group (0.75%) | others |
|  |  | *Lachnospiraceae*-uncultured (0.47%) | others |
|  |  | *Roseburia* (0.45%) | *Roseburia* sp. 831b |
|  | *Ruminococcaceae* (3.34%) | *Faecalibacterium* (1.67%) | others |
|  |  | *Ruminococcaceae* UCG-002 (0.20%) | others |
|  |  | *Ruminiclostridium* 6 (0.20%) | others |

## REFERENCES

Andersson, A. F., Lindberg, M., Jakobsson, H., Backhed, F., Nyren, P., and Engstrand, L. (2008). Comparative analysis of human gut microbiota by barcoded pyrosequencing. *PLoS One* 3, e2836.

Andrews, S. (2010). FastQC: a quality control tool for high throughput sequence data. *Babraham Bioinforma*.

Baker, G. C., Smith, J. J., and Cowan, D. A. (2003). Review and re-analysis of domain-specific 16S primers. *J. Microbiol. Methods* 55, 541-555.

Berlemont, R., Winans, N., Talamantes, D., Dang, H., and Tsai, H. W. (2020). MetaGeneHunt for protein domain annotation in short-read metagenomes. *Sci. Rep.* 10, 7712.

Carr, R., and Borenstein, E. (2014). Comparative analysis of functional metagenomic annotation and the mappability of short reads. *PLoS One* 9, e105776.

Chen, H., Fu, K., Pang, B., Wang, J., Li, H., Jiang, Z., et al. (2020). Determination of uterine bacterial community in postpartum dairy cows with metritis based on 16S rDNA sequencing. *Vet. Anim. Sci.* 10, 100102.

Chen, S., Zhou, Y., Chen, Y., and Gu, J. (2018). Fastp: an ultra-fast all-in-one FASTQ preprocessor. *Bioinformatics* 34, i884-i890.

Dethlefsen, L., Huse, S., Sogin, M. L., and Relman, D. A. (2008). The pervasive effects of an antibiotic on the human gut microbiota, as revealed by deep 16S rRNA sequencing. *PLoS Biol.* 6, e280.

Fierer, N., Hamady, M., Lauber, C. L., and Knight, R. (2008). The influence of sex, handedness, and washing on the diversity of hand surface bacteria. *Proc. Natl. Acad. Sci. U. S. A.* 105, 17994-17999.

Hongoh, Y., Ohkuma, M., and Kudo, T. (2003). Molecular analysis of bacterial microbiota in the gut of the termite *Reticulitermes speratus* (Isoptera; Rhinotermitidae). *FEMS Microbiol. Ecol.* 44, 231-242.

Huse, S. M., Dethlefsen, L., Huber, J. A., Mark Welch, D., Relman, D. A., and Sogin, M. L. (2008). Exploring microbial diversity and taxonomy using SSU rRNA hypervariable tag sequencing. *PLoS Genet.* 4, e1000255.

Juretschko, S., Timmermann, G., Schmid, M., Schleifer, K. H., Pommerening-Röser, A., Koops, H. P. et al. (1998). Combined molecular and conventional analyses of nitrifying bacterium diversity in activated sludge: *Nitrosococcus mobilis* and *Nitrospira*-like bacteria as dominant populations. *Appl. Environ. Microbiol.* 64, 3042-3051.

Keijser, B. J., Zaura, E., Huse, S. M., van der Vossen, J. M., Schuren, F. H., Montijn, R. C., et al. (2008). Pyrosequencing analysis of the oral microflora of healthy adults. *J. Dent. Res.* 87, 1016-1020.

Kim, Y. M., Ahn, C. K., Woo, S. H., Jung, G. Y., and Park, J. M. (2009). Synergic degradation of phenanthrene by consortia of newly isolated bacterial strains. *J. Biotechnol.* 144, 293-298.

Kopylova, E., Noé, L., and Touzet, H. (2012). SortMeRNA: fast and accurate filtering of ribosomal RNAs in metatranscriptomic data. *Bioinformatics* 28, 3211-3217.

Lane, D. J. (1991). 16S/23S rRNA sequencing. Nucleic acid techniques in bacterial systematics. E. Stackebrandt and M. Goodfellow. New York, John Wiley & Sons: 115-175.

Lane, D. J., Pace, B., Olsen, G. J., Stahl, D. A., Sogin, M. L., and Pace, N. R. (1985). Rapid determination of 16S ribosomal RNA sequences for phylogenetic analyses. *Proc. Natl. Acad.* *Sci. U. S. A*. 82, 6955-6959.

Muyzer, G., Teske, A., Wirsen, C. O., and Jannasch, H. W. (1995). Phylogenetic relationships of *Thiomicrospira* species and their identification in deep-sea hydrothermal vent samples by denaturing gradient gel electrophoresis of 16S rDNA fragments. *Arch. Microbiol.* 164, 165-172.

Nossa, C. W., Oberdorf, W. E., Yang, L., Aas, J. A., Paster, B. J., Desantis, T. Z., et al. (2010). Design of 16S rRNA gene primers for 454 pyrosequencing of the human foregut microbiome. *World J. Gastroenterol.* 16, 4135-4144.

Peters, B. A., Wilson, M., Moran, U., Pavlick, A., Izsak, A., Wechter, T., et al. (2019). Relating the gut metagenome and metatranscriptome to immunotherapy responses in melanoma patients." *Genome Med.* 11, 61.

Rodriguez-R, L. M., Gunturu, S., Tiedje, J. M., Cole, J. R., and Konstantinidis, K. T. (2018). Nonpareil 3: fast estimation of metagenomic coverage and sequence diversity. *mSystems* 3, e00039-00018.

Rodriguez, R. L., and Konstantinidis, K. T. (2014). Nonpareil: a redundancy-based approach to assess the level of coverage in metagenomic datasets. *Bioinformatics* 30, 629-635.

Shen, W., Le, S., Li, Y., and Hu, F. (2016). SeqKit: a cross-platform and ultrafast toolkit for FASTA/Q file manipulation. *Plos One* 11, e0163962.

Sogin, M. L., Morrison, H. G., Huber, J. A., Mark Welch, D., Huse, S. M., Neal, P. R., et al. (2006). Microbial diversity in the deep sea and the underexplored "rare biosphere". *Proc. Natl. Acad. Sci. U. S. A.* 103, 12115-12120.

Suzuki, M. T., and Giovannoni, S. J. (1996). Bias caused by template annealing in the amplification of mixtures of 16S rRNA genes by PCR. *Appl. Environ. Microbiol.* 62, 625-630.

Walters, W. A., Caporaso, J. G., Lauber, C. L., Berg-Lyons, D., Fierer, N., and Knight, R. (2011). PrimerProspector: de novo design and taxonomic analysis of barcoded polymerase chain reaction primers. *Bioinformatics* 27, 1159-1161.

Wang, Y., and Qian, P. Y. (2009). Conservative fragments in bacterial 16S rRNA genes and primer design for 16S ribosomal DNA amplicons in metagenomic studies. *PLoS One* 4, e7401.
